# Noble Metal-Enhanced Chemically Sensitized Bi_2_WO_6_ for Point-of-Care Detection of *Listeria monocytogenes* in Ready-to-Eat Foods

**DOI:** 10.3390/foods15020293

**Published:** 2026-01-13

**Authors:** Yong Zhang, Hai Yu, Yu Han, Shu Cui, Jingyi Yang, Bingyang Huo, Jun Wang

**Affiliations:** 1 Department of Physics, Tonghua Normal University, Tonghua 134002, China; yuhai@thnu.edu.cn (H.Y.); hanyu@thnu.edu.cn (Y.H.); shucui@thnu.edu.cn (S.C.); jingyiyang@thnu.edu.cn (J.Y.); 2 College of Food Science and Technology, Guangdong Ocean University, Zhanjiang 524088, China; huoby3@gdou.edu.cn

**Keywords:** Bi_2_WO_6_, noble metal, chemical sensitization, gas sensor, *Listeria monocytogenes*, nondestructive detection

## Abstract

*Listeria monocytogenes* (*LM*) contamination constitutes a paramount global threat to food safety, necessitating the urgent development of advanced, rapid, and non-destructive detection methodologies to ensure food security. This study successfully synthesized Bi_2_WO_6_ nanoflowers through optimized feed ratios of raw materials and further functionalized them with noble metal Au to construct a high-performance Au-Bi_2_WO_6_ composite nanomaterial. The composite exhibited high sensing performance toward acetoin, including high sensitivity (R_a_/R_g_ = 36.9@50 ppm), rapid response–recovery kinetics (13/12 s), and excellent selectivity. Through UV-Vis diffuse reflectance spectroscopy (UV-Vis DRS) and X-ray photoelectron spectroscopy (XPS) characterizations, efficient electron exchange between Au and Bi_2_WO_6_ was confirmed. This electron exchange increased the initial resistance of the material, effectively enhancing the response value toward the target gas. Furthermore, the chemical sensitization effect of Au significantly increased the surface-active oxygen content, promoted gas–solid interfacial reactions, and improved the adsorption capacity for target gases. Compared to conventional turbidimetry, the Au-Bi_2_WO_6_ nanoflower-based gas sensor demonstrates superior practical potential, offering a novel technological approach for non-destructive and rapid detection of foodborne pathogens.

## 1. Introduction

*Listeria monocytogenes* (*LM*) is a Gram-positive, facultative intracellular pathogen that poses significant threats to food safety and public health due to its ability to survive and proliferate under diverse environmental conditions, including refrigeration temperatures, high salinity, and low pH [1]. This pathogen is particularly concerning in ready-to-eat foods, such as dairy products, meat, and leafy vegetables, where it can cause severe infections, including listeriosis, with high mortality rates among immunocompromised individuals, pregnant women, and the elderly [2]. The hazards associated with *LM* are multifaceted, encompassing its biofilm-forming capacity, antimicrobial resistance, and persistence in food-processing environments, which collectively complicate control measures and risk mitigation strategies. Modern food supply chains (such as fresh e-commerce and cold chain logistics) require detection technologies to keep pace with logistics speed [3]. A detection cycle spanning several days leads to product backlog in warehouses, compromising freshness and increasing costs [4]. Therefore, developing rapid methods that yield results within hours or even minutes is a core technological guarantee for achieving efficient and agile supply chain management.

The identification technology using volatile organic compounds (VOCs) as biomarkers has emerged as a approach in the field of microbial detection, offering rapid, non-invasive, and species-specific diagnostic capabilities [5]. During its metabolic processes, *LM* produces unique VOCs, among which 3-hydroxy-2-butanone (3H2B), as the most abundant gas, is recognized as a specific biomarker for *LM* [6]. This compound enables the detection of food contamination by *LM*, providing a valuable tool for food safety monitoring. The Micro-Electro-Mechanical Systems (MEMS) gas sensor is a resistive sensor that operates on the principle of interactions between target gases and the surface of sensitive materials, causing a change in the material’s electrical resistance to enable the conversion of chemical signals into electrical signals [7]. In application scenarios such as food safety and environmental monitoring, this technology achieves an optimal balance between miniaturization, cost-effectiveness, and detection performance.

Bismuth tungstate (Bi_2_WO_6_), owing to its distinctive structural and electronic properties, has emerged as a promising semiconductor gas-sensing material with broad application prospects, enabling selective and sensitive detection of VOCs and other target gases [8]. As a mixed metal oxide, Bi_2_WO_6_ features tunable surface acidity and oxygen vacancy concentration, which are critical for gas adsorption and redox reactions [9,10]. The metal-oxygen bond energy of the material directly governs its surface reactivity, allowing tailored interactions with specific analytes [11]. For instance, the presence of Bi^3+^ cations in the lattice enhances the material’s affinity for VOCs, while the WO_4_^2−^ framework facilitates stable charge transfer during the gas-sensing process [12]. This synergistic effect between bismuth and tungsten oxides confers superior selectivity to Bi_2_WO_6_ compared to single metal oxides such as ZnO or SnO_2_ [13,14].

A key advantage of Bi_2_WO_6_ lies in its catalytic activity, which can be further enhanced through functionalization with noble metals [15]. These additives promote the oxidation of reducing gases at lower operating temperatures, thereby improving sensor response time and energy efficiency [16]. Au serves as a highly efficient sensitizer for resistive semiconductor gas-sensing materials, primarily through the catalytic activity of its nanoparticles to promote the dissociation of target gases [17,18]. Hyodo et al. [19] systematically investigated the influence of Au-modified Pt sensing electrodes on the hydrogen sensing performance and mechanisms in TiO_2_-based diode-type hydrogen sensors. The study revealed that Au nanoparticles effectively modulated the activity of surface oxygen species, enhanced hydrogen dissociation and adsorption, and significantly improved the response performance of TiO_2_-based diode-type hydrogen sensors, particularly demonstrating high performance in ambient air environments. Concurrently, the formation of a Schottky barrier at the metal–semiconductor interface enables dynamic modulation of barrier height, thereby amplifying the resistive response. Jiang et al. [17] demonstrated that the formation of a Schottky contact between Au and K_2_W_4_O_13_ effectively modulates electron transport behavior, enhances the electron depletion layer, and increases the baseline resistance of the sensor in ambient air. This strategic surface engineering approach achieved gas sensing performance characterized by low-temperature operation, high responsiveness, and dual selectivity. This synergistic mechanism allows the sensor to achieve high sensitivity and selectivity for specific gases at lower operating temperatures, effectively reducing power consumption and extending device lifespan. The integrated catalytic–electronic enhancement strategy exemplifies a sophisticated approach to optimizing gas sensor performance, balancing operational efficiency with detection precision in advanced sensing applications [20,21].

This study successfully prepared Bi_2_WO_6_ nanoflower structures through optimized raw material ratios and further functionalized them with noble metal Au, constructing high-performance Au-Bi_2_WO_6_ composite nanomaterials. The research systematically employed advanced characterization techniques such as UV-Vis DRS and XPS to elucidate the chemical sensitization mechanism of Au. Experiments confirmed that Au modification significantly enhances the content of reactive oxygen species on the material surface, optimizes gas–solid interfacial reaction kinetics, and effectively improves the adsorption capacity for target gases. The developed Au-Bi_2_WO_6_ nanoflower gas sensor demonstrated outstanding sensing performance in detecting *LM* MVOCS, including high sensitivity (R_a_/R_g_ = 36.9@50 ppm), rapid response/recovery kinetics (13/12 s), and excellent selectivity. Compared to traditional turbidimetric detection methods, this sensor exhibits significant advantages in non-destructive and rapid detection of foodborne pathogens. Its unique sensing mechanism and performance characteristics provide an innovative technological solution for food safety monitoring, offering broad prospects for industrial applications.

## 2. Materials and Methods

### 2.1. Reagents and Instruments

All analytical-grade chemicals and solvents were purchased and used without further purification. H_2_SO_4_, oleylamine, n-hexane, and anhydrous ethanol were bought from Sinopharm Chemical Reagent Co., Ltd., (Shanghai, China). Bi(NO_3_)_3_·5H_2_O, Na_2_WO_4_·2H_2_O, HAuCl_4_·3H_2_O, and Na_2_SO_4_ were gained from Sigma-Aldrich, (St. Louis, MO, USA). The serotype 1/2a *Listeria monocytogenes* strain CMCC54002, the *Escherichia coli* strains CMCC(B)44102/44103, and the *Staphylococcus aureus* strain CMCC(B) 26003, obtained from Shanghai Beikangnuo Biotechnology Co., Ltd. (Shanghai, China), represent one of the most globally prevalent serotypes implicated in foodborne listeriosis outbreaks. This pathogenic isolate was maintained in a −80 °C glycerol stock and routinely subculture for experimental activation before utilization in study procedures.

At 25 °C, the crystal structure of the materials was characterized via X-ray diffraction ((XRD; Rigaku, Tokyo, Japan)) with a copper target (wavelength λ = 1.5418 Å). Scanning electron microscopy (SEM, SU5000; Hitachi, Tokyo, Japan) and high-resolution transmission electron microscopy (HRTEM; JEOL JEM-2011; JEOL Ltd., Tokyo, Japan) were employed to examine the microstructure. A UV-Vis spectrophotometer equipped with an integrating sphere attachment (UV-vis 2550; Shimadzu Corporation, Kyoto, Japan) was used to obtain the solid-state UV-Vis diffuse reflectance spectra. The chemical composition was analyzed by X-ray photoelectron spectroscopy (XPS; PHI-5000CESCA; ULVAC-PHI, Chigasaki, Japan). Moreover, the specific surface area was determined using the Brunauer-Emmett-Teller (ASAP2460; Micromeritics Instrument Corporation, Norcross, GA, USA) method, with N_2_ serving as the adsorption–desorption medium.

### 2.2. Synthesis of Bi_2_WO_6_ with Different Morphologies

Dissolve 0.2425 g of Bi(NO_3_)_3_·5H_2_O and 0.0824 g of Na_2_WO_4_·2H_2_O in 40 mL of deionized water. Then add a certain amount of Na_2_SO_4_ to the above mixture. After adjusting the pH value to 2, transfer the mixture to a reaction kettle and keep it at 180 °C for 24 h. Collect the final product by centrifugation, wash with deionized water and ethanol, and dry at 60 °C for 12 h. Among them, the sample with 1 g of Na_2_SO_4_ added is referred to as Bi_2_WO_6_-1, the sample with 3 g of Na_2_SO_4_ added is referred to as Bi_2_WO_6_-2, and the sample with 5 g of Na_2_SO_4_ added is referred to as Bi_2_WO_6_-3.

### 2.3. Synthesis of Au Nanoparticles (NPs)

Firstly, add 10 mL of oleylamine solution into a three-necked flask and heat it in an oil bath to 110 °C, reflux for 10 min. Then, add 0.059 g of HAuCl_4_·3H_2_O, continue heating up to 220 °C for 10 min, and then continue heating up to 240 °C for 10 min. After natural cooling, centrifuge and wash with a mixture of n-hexane and ethanol at least 5 times, followed by drying at 60 °C for 12 h.

### 2.4. Synthesis of Au-Bi_2_WO_6_

Disperse 0.1 g of Bi_2_WO_6_ in 15 mL of deionized water and sonicate. Then, add different amounts of Au nanoparticles to the suspension and continue ultrasonication for 30 min. The obtained Au-Bi_2_WO_6_ nanocomposite was collected after drying at 60 °C. Based on the loading amount (mass percentage) of silver nanoparticles, these samples were labeled as X% Au-Bi_2_WO_6_ (X = 0.5, 1.0, 1.5).

### 2.5. Practical Sample Analysis of LM

In the initial step of the procedure, bacterial activation was achieved through overnight incubation in BHI broth. A sequential ten-fold dilution of the bacterial suspension was prepared, resulting in concentrations ranging from 10^1^ to 10^7^ CFU mL^−1^. From each dilution, 150 μL was dispensed into a 40 mL headspace vial and incubated at 37 °C with agitation set to 150 rpm. The growth profiles of cultures starting from varying cell densities were tracked through periodic sampling every two hours, followed by detection using Au-Bi_2_WO_6_ sensors. In parallel, to assess bacterial concentration, 200 μL samples taken from the same dilution series were placed into a 96-well plate for optical density measurement at 600 nm (OD_600_). For comparison, *Staphylococcus aureus* and *Escherichia coli* were similarly cultured and evaluated under identical experimental conditions.

### 2.6. Method Validation

The linear range and sensitivity were determined by performing linear regression between the response value (S) and the concentration (c) to obtain the equation: S = k × c + b. The linear range was selected as the concentration interval where the coefficient of determination (R^2^) exceeded 0.99 (or 0.995), indicating a strong linear relationship. The limit of detection (LOD) and limit of quantitation (LOQ) were calculated using the baseline signal measured under air multiple times. The standard deviation (σ) of the blank response was computed, and LOD was defined as 3 × σ/k (corresponding to a signal-to-noise ratio S/N ≈ 3), while LOQ was defined as 10 × σ/k (S/N ≈ 10), where k represents the slope from the linear regression. The resulting LOD and LOQ were expressed in concentration units (ppm or ppb). Precision was assessed through repetitive experiments at a fixed concentration. Response values (S_1_, S_2_,…, S_n_) were measured n times, and the mean (S_avg_) and standard deviation (SD) of the dataset were calculated. The relative standard deviation (RSD%) was computed using the formula: RSD% = (SD/S_avg_) × 100%. The selectivity coefficient was determined as the ratio of the response value to the target gas versus the response value to an interfering gas, providing a measure of the method’s specificity. All procedures were conducted in the past tense, reflecting completed experimental actions.

## 3. Results

### 3.1. Characterizations of Materials

Morphology control of MOS has emerged as a pivotal strategy for tailoring their physicochemical properties [22]. In this study, three distinct morphologies of Bi_2_WO_6_ were synthesized by precisely modulating the dosage of Na_2_SO_4_, which establishes an ionic environment that governs crystal growth kinetics and orientation [23]. At lower Na_2_SO_4_ concentrations (2 g), irregular two-dimensional (2D) nanosheets are formed (Figure S1a,d). Under optimal conditions (3 g), a well-defined three-dimensional (3D) nanoflower architecture emerges, composed of ordered nanosheet stacking (Figure S1b,e). Conversely, excessive Na_2_SO_4_ (5 g) drastically elevates the ionic strength of the reaction system, disrupting the orderly self-assembly of nanosheets and resulting in irregularly stacked 2D nanosheet-based nanospheres (Figure S1c,f). To elucidate the crystal structure of the synthesized sensitive materials, X-ray diffraction (XRD) analysis was conducted on the three Bi_2_WO_6_ samples. As illustrated in Figure S2, all diffraction patterns exhibit complete correspondence with the orthorhombic Bi_2_WO_6_ phase (PDF card #75-1126), with no detectable impurity peaks confirming the high phase purity of the synthesized materials [24]. Based on the analysis of nitrogen adsorption–desorption isotherms (Figure S3a), all three synthesized Bi_2_WO_6_ samples exhibit Type IV isotherms with H3 hysteresis loops, which are characteristic signatures of mesoporous materials [25]. This finding, corroborated by the pore size distribution data presented in Figure S3b, confirms that the mesoporous nature of these materials originates from the slit-shaped pores formed by the stacking of nanosheet building blocks [26]. Analysis of the specific surface area and pore volume comparison chart (Figure S3c) reveals that the Bi_2_WO_6_-2 sample exhibits the highest specific surface area (20.3 m^2^/g) and largest pore volume (0.085 cm^3^/g) among the synthesized materials. This superior textural property indicates enhanced availability of active sites and elevated gas adsorption capacity, which are critical parameters for optimizing performance in gas sensing applications [27]. Quartz crystal microbalance (QCM) adsorption–desorption experiments demonstrate that Bi_2_WO_6_ with a higher specific surface area exhibits enhanced adsorption capacity for 3H2B and demonstrates the fastest adsorption kinetics among the tested samples [28]. Based on the experimental validation of superior textural properties and enhanced adsorption kinetics, Bi_2_WO_6_ is selected as the host material for subsequent functionalization via loading processes.

**Figure 1:**
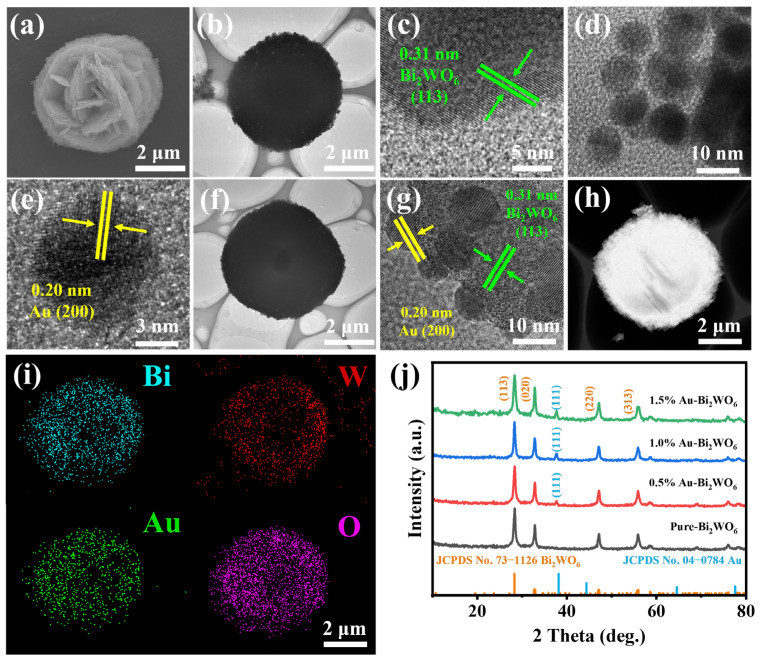
(**a**) SEM, (**b**) TEM, and (**c**) HRTEM images of pure Bi_2_WO_6_, (**d**) TEM and (**e**) HRTEM image of Au nanoparticles, (**f**) TEM and (**g**) HRTEM images of 1.0% Au-Bi_2_WO_6_, (**h**,**i**) STEM-EDS elemental mapping images of 1.0% Au-Bi_2_WO_6_, (**j**) XRD patterns of pure Bi_2_WO_6_, 0.5% Au-Bi_2_WO_6_, 1.0% Au-Bi_2_WO_6_, and 1.5% Au-Bi_2_WO_6_.

To further enhance the gas-sensing performance of pristine Bi_2_WO_6_ nanoflowers, a controlled modification process was employed to functionalize the material with Au NPs. The transmission electron microscopy (TEM) image (Figure 1b) corroborated by SEM results (Figure 1a), confirms that Bi_2_WO_6_ nanoflowers exhibit a complete flower-like morphology with excellent dispersibility. Further high-resolution transmission electron microscopy (HRTEM) analysis (Figure 1c) reveals distinct lattice fringes, and the measured interplanar spacing of 0.31 nm precisely matches the standard spacing of the (113) crystal plane in orthorhombic Bi_2_WO_6_ [29]. This correspondence is further validated by the exact alignment with the characteristic peak position of the (113) plane in the XRD pattern (Figure 1j) [30]. TEM images (Figure 1d,e) demonstrate, through morphological characterization, that Au NPs exhibit uniform spherical morphology and excellent dispersity. HRTEM analysis further reveals distinct lattice fringes with a precisely measured interplanar spacing of 0.200 nm. This value aligns perfectly with both the standard lattice parameter of metallic Au and the theoretical interplanar spacing of the (200) crystal plane, confirming atomic-scale structural integrity [31]. TEM characterization of the Au NPs-functionalized sample (Figure 1f,g) shows uniform dispersion of Au NPs across the nanoflower surface. Energy-dispersive X-ray spectroscopy (EDS) mapping (Figure 1h,i) further validates the homogeneous spatial distribution of Bi, W, O, and Au elements, providing conclusive evidence for the successful integration of Au NPs within the nanoflower architecture [32]. Figure S6 and Table S1 further demonstrate the successful loading of Au with a loading capacity of 1%.

**Figure 2:**
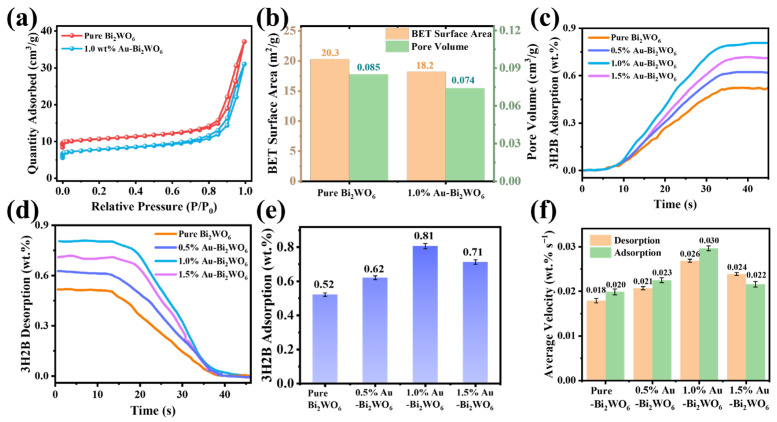
(**a**) Nitrogen adsorption–desorption isotherms, (**b**) BET surface area and pore volume of pure Bi_2_WO_6_ and 1.0% Au-Bi_2_WO_6_, and (**c**–**f**) 3H2B adsorption performance test of pure Bi_2_WO_6_, 0.5% Au-Bi_2_WO_6_, 1.0% Au-Bi_2_WO_6_, and 1.5% Au-Bi_2_WO_6_.

BET analysis demonstrates that the loading of Au nanoparticles did not significantly alter the specific surface area and pore volume characteristics of the material. As observed in Figure 2a, both samples exhibit distinct Type IV isotherms with H3 hysteresis loops, indicating consistent mesoporous structural features [33]. Quantitative calculations demonstrate that after Au loading, the specific surface area decreases by approximately 2.1 m^2^ g^−1^, while the pore volume reduces by about 0.011 cm^3^ g^−1^. This marginal reduction is likely attributed to the occupation and partial blockage of mesopores and internal cavities by Au nanoparticles [34]. Figure 2c–f conclusively demonstrate that Au NPs decoration significantly enhances both the adsorption capacity and kinetic uptake rate of the target gas 3H2B across all tested compositions, and the 1.0 wt.% Au-Bi_2_WO_6_ exhibits the optimal performance enhancement.

**Figure 3:**
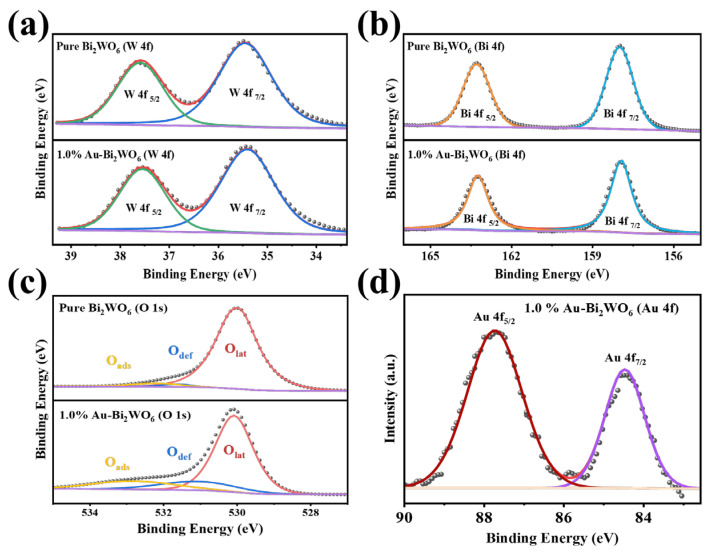
(**a**) W 4f, (**b**) Bi 4f, (**c**) O 1s, and (**d**) Au 4f high-resolution XPS spectrum of pure Bi_2_WO_6_ and 1.0% Au-Bi_2_WO_6_.

To elucidate the influence of Au loading on the chemical states of Bi_2_WO_6_ and the evolution of oxygen species, X-ray photoelectron spectroscopy (XPS) analyses were conducted. The results revealed that both pristine and Au-decorated Bi_2_WO_6_ exhibited distinct characteristic peaks corresponding to W 4f_7/2_ and W 4f_5/2_ (Figure 3a), as well as Bi 4f_7/2_ and Bi 4f_5/2_ (Figure 3b), with no discernible shifts in peak positions post-Au loading [35]. Furthermore, the O 1s XPS spectra (Figure 3c) for both samples displayed three deconvoluted peaks assigned to lattice oxygen (O_lat_), defect oxygen (O_def_), and adsorbed oxygen (O_ads_). Quantitative analysis of the fitted peaks demonstrated a significant enhancement in the concentrations of O_def_ and O_ads_ following Au loading [36]. The increased O_def_ and O_ads_ content amplifies the number of active sites on the material surface, providing more adsorption sites for gas molecules [37]. Additionally, the presence of Au was conclusively verified by the two characteristic peaks corresponding to Au 4f_7/2_ and Au 4f_5/2_ in Figure 3d, further confirming the successful integration of Au nanoparticles.

### 3.2. Gas Sensitivity Performance

Systematic characterization of the gas sensing performance of synthesized pure Bi_2_WO_6_ was first conducted. Figure S5a demonstrates the gas-sensing response of Bi_2_WO_6_-1, Bi_2_WO_6_-2, and Bi_2_WO_6_-3 samples toward 50 ppm 3H2B across a temperature range of 150–330 °C. The optimal operating temperature for all samples was identified as 270 °C, with Bi_2_WO_6_-2 exhibiting the highest response magnitude. Linear calibration curves were established for all three samples across varying concentrations of 3H2B, as validated in Figure S5b,c, confirming excellent linearity and reproducibility. Selectivity assessments (Figure S5d) revealed that while all samples demonstrated favorable selectivity toward 3H2B over common interfering gases, further optimization of the microstructure and surface chemistry is required. Building on these findings, the highest-performing Bi_2_WO_6_-2 substrate was selected for Au NPs functionalization.

**Figure 4:**
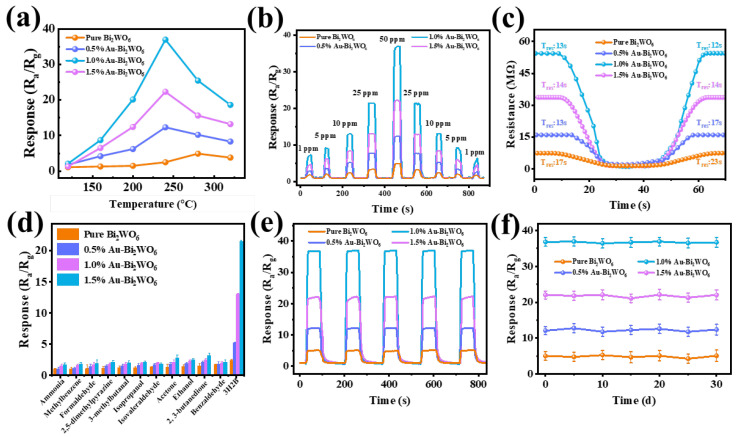
Gas sensing performance of pure Bi_2_WO_6_, 0.5% Au-Bi_2_WO_6_, 1.0% Au-Bi_2_WO_6_, and 1.5% Au-Bi_2_WO_6_: (**a**) response to 50 ppm 3H2B (120–320 °C) at different working temperatures, (**b**) dynamic response of 3H2B concentration (1–50 ppm), (**c**) response/recovery time (50 ppm 3H2B), (**d**) selectivity of 25 ppm 3H2B and 50 ppm other interfering gases, (**e**) reproducibility and (**f**) long-term stability at 50 ppm 3H2B.

Figure 4a illustrates the response curves of pure Bi_2_WO_6_ and Au-loaded Bi_2_WO_6_-based sensors with varying Au loading contents toward 50 ppm 3H2B across different operating temperatures. Comparative analysis reveals that Au loading reduces the optimal operating temperature from 270 °C to 240 °C, indicating that Au sensitization effectively lowers the chemical reaction barrier and significantly accelerates the reaction kinetics. The highest response value toward 3H2B is achieved by the 1.0 wt.% Au-Bi_2_WO_6_ sensor, exhibiting a 7.5-fold improvement in performance compared to pristine Bi_2_WO_6_. This enhancement is attributed to the unique chemical sensitization effect of noble metal Au, which facilitates charge transfer and catalytic activity [38]. Furthermore, as demonstrated in Figure 4b and Figure S7a, both pure and Au-loaded Bi_2_WO_6_ sensors demonstrate excellent dynamic responses toward varying concentrations of 3H2B, with a remarkably low detection limit of 93 ppb (Table S2). The tables showing the LOD, LOQ, RSD, and linearity range for different materials are as follows in Tables S2 and S3. From the table, it can be seen that 1.0% Au-Bi_2_WO_6_ has the lowest theoretical LOD and the smallest LOQ, and all samples show a good linear relationship within the range of 0–50 ppm. The above results clearly indicate that the developed method is reliable, sensitive, and suitable for its intended application. The core performance metrics that determine the practical application efficacy of sensors include response-recovery characteristics, selectivity, interference resistance, repeatability, long-term stability, and moisture resistance [39]. As demonstrated in Figure 4c, the 1.0 wt.% Au-Bi_2_WO_6_ sensor exhibits high dynamic response characteristics in target gas detection. Its response time and recovery time are optimized to 13/12 s, respectively, significantly shorter than those of conventional material systems, showcasing superior rapid dynamic response advantages. To evaluate selectivity and interference resistance, interference gases such as benzaldehyde and 2,3-butanedione—organic compounds abundantly exhaled during the metabolic growth of *LM*—as well as common gases in practical applications, were tested. The results, shown in Figure 4d and Figure S7b, confirm that the 1.0 wt.% Au-Bi_2_WO_6_ sensor possesses excellent selectivity and anti-interference capabilities, demonstrating robust molecular recognition specificity. This ensures accurate target molecule identification in complex gas environments, minimizing false-positive/negative misjudgments [2]. Moreover, 1.0% Au-Bi_2_WO_6_ has the highest selectivity coefficient for different interfering gases (Figure S8). The structure of 3H2B enables it to be more readily oxidized on the Au/Bi_2_WO_6_ surface at our operating temperature, resulting in a stronger electronic response than less reactive analogs.

Regarding repeatability and long-term stability, cyclic tests over five consecutive cycles (Figure 4e) and a 30-day long-term stability assessment (Figure 4f) verify its outstanding cyclic reversibility and operational stability under prolonged use. Further calculations of the intraday and diurnal variations of the material demonstrated its stability and good reproducibility (Tables S4 and S5). These superior stability metrics effectively mitigate the risk of performance degradation and irreversible adsorption-induced failures commonly observed in traditional chemiresistors, thereby ensuring measurement reliability in continuous monitoring scenarios. As a critical indicator of environmental adaptability, moisture resistance was systematically evaluated through humidity gradient experiments (40–80% RH) to assess the impact of water molecules on sensor performance. As shown in Figure S7c, although high-humidity environments induce competitive adsorption between water molecules and target gas molecules on the sensing material surface, leading to a slight attenuation in response values, the maximum fluctuation range is strictly controlled within ±5%. This controlled humidity tolerance mechanism enables stable sensor operation in high-humidity environments. Such capability significantly expands the application scope to humidity-sensitive domains, including food microbiological detection, agricultural crop monitoring, and pharmaceutical storage condition verification, where traditional sensors often suffer from humidity-induced signal drift and baseline instability [40].

In summary, through multi-dimensional performance optimization, the 1.0 wt.% Au-Bi_2_WO_6_ sensor achieves practical requirements in response kinetics, interference resistance, and environmental adaptability. It provides a highly reliable sensing solution for the rapid detection of pathogenic microorganisms such as *LM*, demonstrating significant technological advantages and broad application prospects.

### 3.3. Gas-Sensing Mechanism

**Figure 5:**
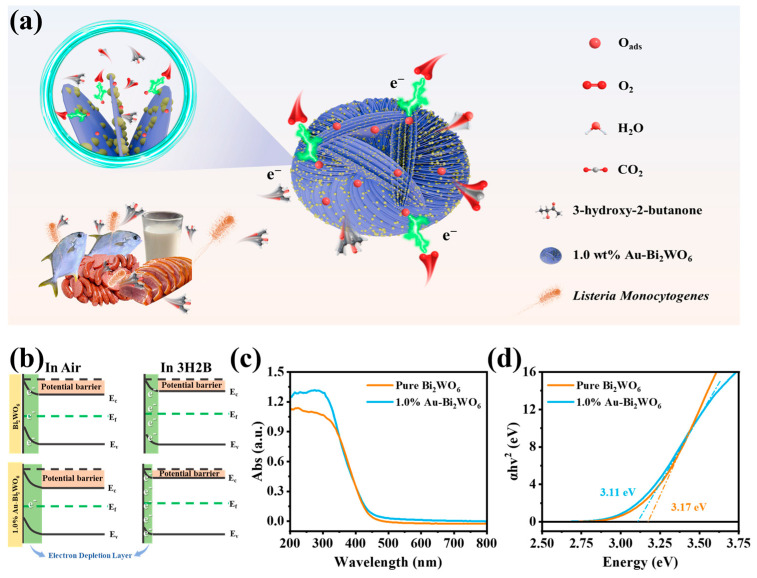
(**a**) Schematic illustration of the 3H2B sensing mechanism of 1.0% Au-Bi_2_WO_6_-based sensor (**b**) the electronic structural change, (**c**) UV-vis DRS, and (**d**) Tauc curve of pure Bi_2_WO_6_ and 1.0% Au-Bi_2_WO_6_.

Multiple theoretical models exist to explain the operating mechanisms of semiconductor gas sensors, among which the surface depletion model is widely recognized as a fundamental framework for interpreting the sensing mechanism of the 1.0 wt.% Au-Bi_2_WO_6_ gas sensor [41]. This model constitutes one of the established theoretical frameworks for elucidating the working principles of oxide semiconductor gas sensors. The core of the surface depletion model lies in the redox reactions occurring at the surface of oxide semiconductor sensing materials. Upon exposure to air, oxygen molecules adsorb onto the surface, where they capture free electrons from the semiconductor’s near-surface region to form chemisorbed oxygen species, thereby creating an electron depletion layer at the semiconductor surface [18]. The model posits that the thickness of this depletion layer is determined by the quantity of adsorbed oxygen molecules and the electron concentration at the semiconductor surface. Under equilibrium conditions, the depletion layer thickness and the adsorbed oxygen quantity reach a dynamic balance. When a target gas interacts with the sensor, it reacts with chemisorbed oxygen ions, releasing trapped electrons back into the semiconductor [19]. This process is predicted by the model to reduce the depletion layer thickness, increase semiconductor conductivity, and decrease electrical resistance (Figure 5a).

The enhanced gas-sensing performance following noble metal Au functionalization is primarily attributed to experimental observations of both electronic and chemical sensitization effects of Au in the 1.0 wt.% Au-Bi_2_WO_6_ sensor. The electronic sensitization effect originates from the work function difference between the noble metal Au and Bi_2_WO_6_. When Au is deposited onto the MOS surface, electron transfer occurs between Au and the MOS due to their differing work functions until Fermi level equilibrium is achieved (Figure 5b). Solid-state UV-Vis diffuse reflectance spectroscopy and derived Tauc plots for Bi_2_WO_6_ and 1.0 wt.% Au-Bi_2_WO_6_ (Figure 5c,d) demonstrates that Au nanoparticle functionalization reduces the material’s bandgap from 3.17 eV to 3.11 eV, confirming effective electron exchange between Au and Bi_2_WO_6_ [13]. This electron redistribution is hypothesized to modify carrier transport properties, thereby influencing gas sensor performance. Specifically, the electronic sensitization is suggested to optimize resistance changes, enhancing sensitivity and selectivity toward target gases. The chemical sensitization effect of Au is manifested through its superior catalytic properties, which facilitate oxygen dissociation on the material surface to generate more reactive chemisorbed oxygen species [20]. These species, distributed across the MOS surface via spillover effects, are experimentally observed to increase reaction opportunities with target gas molecules, thereby improving sensitivity, reducing operating temperature, and accelerating response/recovery kinetics [42].

### 3.4. Practical Application

**Figure 6:**
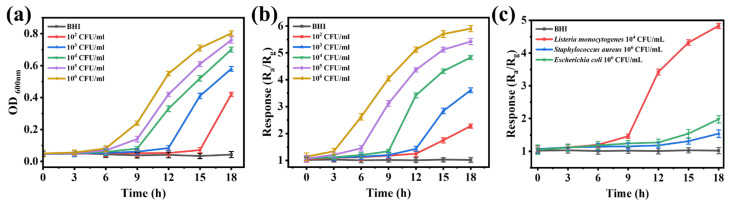
(**a**) The OD values of the various concentrations of *LM*, (**b**) the response value of the 1.0% Au-Bi_2_WO_6_-based sensors to various concentrations of *LM*, (**c**) the responses of the 1.0% Au-Bi_2_WO_6_-based sensors to 10^6^ CFU mL^−1^ Staphylococcus aureus and Escherichia coli and 10^4^ CFU mL^−1^ *LM*.

The real-time monitoring of specific MVOCs or metabolic markers released during the growth and metabolism of *LM* in brain heart infusion (BHI) broth enabled a systematic evaluation of the detection performance and response mechanism of a 1.0% Au-Bi_2_WO_6_ nanocomposite sensor in this study. To comprehensively validate the reliability and accuracy of detection results, traditional turbidimetry (OD_600_) was simultaneously employed to track bacterial growth kinetics (Figure 6a,b), with comparative analyses conducted. The results demonstrated that the sensor’s response curve not only precisely replicated the typical growth cycle characteristics of *LM*—exhibiting a flat initial lag phase, followed by a rapid exponential growth phase, and ultimately transitioning to a stationary phase—but also showed high correlation between response signals and bacterial metabolic activity [43]. This reflects the sensitive adsorption and interfacial recognition process of 1.0% Au-Bi_2_WO_6_ materials toward bacterial metabolites.

Notably, the nanocomposite sensor exhibited significant advantages in detection speed. Compared to turbidimetry, which typically requires bacterial concentrations of 10^6^–10^7^ CFU/mL to determine growth status, this sensor captured faint metabolic signals at early culture stages, substantially advancing the detection window. In low-bacterial-concentration challenge experiments, even at an initial inoculum concentration of 10^2^ CFU/mL, the sensor delivered a response signal with an excellent signal-to-noise ratio, well below the detection limits of conventional methods. This sensitivity fully meets the requirements of international food safety standards [3]. Furthermore, the sensor demonstrated favorable selectivity against common foodborne pathogens, including *Escherichia coli* and *Staphylococcus aureus*, underscoring its substantial potential for early warning and rapid detection of *LM* contamination in food matrices.

In a concise quantitative comparison with recently reported LM detection methods (e.g., biosensors, electronic nose systems, optical sensors), the method presented in this study exhibits advantages in response time, detection limit, or non-destructive capability (Table S6). This multidimensional performance optimization positions the 1.0% Au-Bi_2_WO_6_ nanocomposite sensor as a highly reliable sensing solution for detecting pathogenic microorganisms, offering distinct technological advantages and broad application prospects in the field of food safety monitoring. This proof-of-concept study was conducted in a standard BHI medium to establish selective detection of 3H2B under controlled and reproducible metabolite production conditions. In real food matrices, complex composition, background volatiles, competitive microflora, and variable storage conditions may significantly influence microbial metabolism and sensor performance, potentially altering the VOC profile and affecting detection sensitivity and selectivity. Therefore, future work should focus on validating and optimizing the sensor system in specific, complex real-world food environments to advance this technology from a laboratory concept to a practical monitoring tool.

## 4. Conclusions

This study presents the synthesis of a nanosheet-assembled Bi_2_WO_6_ nanoflower architecture through an optimized synthesis route, followed by surface functionalization with Au to construct an Au-Bi_2_WO_6_ composite nanomaterial. The Au-Bi_2_WO_6_ nanoflower-based sensor shows improved sensing characteristics, including a response (R_a_/R_g_) of 36.9 toward 50 ppm 3H2B, fast response/recovery times (13/12 s), and good selectivity. By employing ultraviolet-visible UV-Vis DRS and XPS, the chemical sensitization effect of Au was investigated. Results confirm efficient electron exchange between Au and Bi_2_WO_6_, leading to an increased initial resistance. Meanwhile, Au modification promotes the surface concentration of reactive oxygen species, optimizes gas–solid interfacial reaction kinetics, and improves the adsorption capacity for the target gas. Compared with conventional turbidity-based methods, this sensor offers a promising approach for non-destructive and rapid detection of foodborne pathogens. This work reports a non-destructive monitoring of foodborne bacteria, which may contribute to food safety applications.

## Data Availability

The original contributions presented in the study are included in the article/Supplementary Materials, further inquiries can be directed to the corresponding authors.

## Supplementary Materials

The following supporting information can be downloaded at: https://www.mdpi.com/article/10.3390/foods15020293/s1, Figure S1: SEM images of Bi_2_WO_6_ synthesized by adding different Na_2_SO_4_. (a) 1 g, (b) 3 g, and (c) 5 g; Figure S2: XRD patterns of Bi_2_WO_6_ with different Na_2_SO_4_ additions; Figure S3: (a) Nitrogen adsorption–desorption isotherms, (b) pore size distributions, and (c) BET surface area and Pore volume of pristine Bi_2_WO_6_-1, Bi_2_WO_6_-2, and Bi_2_WO_6_-3; Figure S4: QCM adsorption desorption test of 3H2B on Bi_2_WO_6_ synthesized with different Na_2_SO_4_ addition amounts. (a) Adsorption curve, (b) desorption curve, (c) maximum adsorption capacity, and (d) adsorption/desorption rate; Figure S5: Gas-sensing properties of Bi_2_WO_6_ synthesized based on different Na_2_SO_4_ addition amounts for 3H2B. (a) The response of the sensor to 50 ppm 3H2B at different working temperatures (120–320 °C). (b) Dynamic response curves of sensors to different concentrations of 3H2B (1–50 ppm) at 280 °C. (Cc) Linear relationship between gas sensor response at 280 °C and 3H2B concentration (d). The selectivity of the sensor towards 50 ppm 3H2B and other interfering gases at 280 °C; Figure S6: EDS spectra of 1.0% Au-Bi_2_WO_6_ material; Figure S7: Gas sensing performance: (a) linear relationship between response and 3H2B concentration (1~50 ppm) of pure Bi_2_WO_6_, 0.5% Au-Bi_2_WO_6_, 1.0% Au-Bi_2_WO_6_, and 1.5% Au-Bi_2_WO_6_. (b) Response to mixed gases containing 25 ppm 3H2B and 25 ppm other interfering gases, and (c) response to 50 ppm 3H2B at different humidity values of 1.0% Au-Bi_2_WO_6_; Figure S8: Selectivity coefficients of different materials for different interfering gases; Table S1: Atomic proportion of 1.0% Au-Bi_2_WO_6_ material; Table S2: LOD, LOQ, RSD and Linearity range for different materials; Table S3: Linear relationship, R^2^, and σ for different materials; Table S4: Intra-day variation for different materials; Table S5: Inter-day variation for different materials; Table S6: Comparison Table of Different Detection Methods for Listeria monocytogenes.

## Abbreviations

The following abbreviations are used in this manuscript:

BET	Brunauer–Emmett–Teller
Bi_2_WO_6_	Bismuth tungstate
*LM*	*Listeria monocytogenes*
LOD	Limit of Detection
LOQ	limit of quantitation
MVOC	Microbial volatile organic compound
MEMS	Micro-Electro-Mechanical Systems
NPs	Nanoparticles
OD_600_	Optical density measurement at 600 nm
O_lat_	Lattice oxygen
O_ads_	Defect oxygen
O_def_	Adsorbed oxygen
QCM	Quartz crystal microbalance
RSD	Relative standard deviation
SD	Standard deviation
SEM	Scanning electron microscopy
TEM	Transmission electron microscopy
UV-Vis DRS	UV-Vis diffuse reflectance spectroscopy
XPS	X-ray photoelectron spectroscopy
XRD	X-ray diffraction
3H2B	3-hydroxy-2-butanone
