# Supplementary material for: Noble Metal-Enhanced Chemically Sensitized Bi2WO6 for Point-of-Care Detection of Listeria monocytogenes in Ready-to-Eat Foods
Source: Foods. 2026 Jan 13;15(2):293. doi: 10.3390/foods15020293 (PMC12840556; doi:10.3390/foods15020293)
Supplement: Supplementary file 1 [file foods-15-00293-s001.zip › foods-4056706-supplementary.pdf]

Supplementary Material

Noble Metal-Enhanced Chemically Sensitized Bi_2WO_6 for Point-of-Care Detection of *Listeria monocytogenes* in Ready-to-Eat Foods

Figure and Table caption:

Figure S1. SEM images of Bi_2WO_6 synthesized by adding different Na_2SO_4. (a) 1g, (b) 3g, and (c) 5g.

Figure S2. XRD patterns of Bi_2WO_6 with different Na_2SO_4 additions.

Figure S3. (a) Nitrogen adsorption-desorption isotherms, (b) pore size distributions, and (c) BET surface area and Pore volume of pristine Bi_2WO_6-1, Bi_2WO_6-2, and Bi_2WO_6-3.

Figure S4. QCM adsorption desorption test of 3H2B on Bi_2WO_6 synthesized with different Na_2SO_4 addition amounts. (a) Adsorption curve, (b) desorption curve, (c) maximum adsorption capacity, and (d) adsorption/desorption rate.

Figure S5. Gas-sensing properties of Bi_2WO_6 synthesized based on different Na_2SO_4 addition amounts for 3H2B. (a) The response of the sensor to 50 ppm 3H2B at different working temperatures (120-320 ℃). (b) Dynamic response curves of sensors to different concentrations of 3H2B (1-50 ppm) at 280 ℃. (C) Linear relationship between gas sensor response at 280 ℃ and 3H2B concentration. (d) The selectivity of the sensor towards 50 ppm 3H2B and other interfering gases at 280 ℃.

Figure S6: EDS spectra of 1.0% Au-Bi_2WO_6 material.

Figure S7. Gas sensing performance: (a) linear relationship between response and 3H2B concentration (1~50 ppm) of pure Bi_2WO_6, 0.5% Au-Bi_2WO_6, 1.0% Au-Bi_2WO_6, and 1.5% Au-Bi_2WO_6. (b) Response to mixed gases containing 25 ppm 3H2B and 25 ppm other interfering gases, and (c) response to 50 ppm 3H2B at different humidity values of 1.0% Au-Bi_2WO_6.

Figure S8: Selectivity coefficients of different materials for different interfering gases.

Table S1: Atomic proportion of 1.0% Au-Bi_2WO_6 material.

Table S2: LOD, LOQ, RSD, and Linearity range for different materials.

Table S3: Linear relationship, R^2, and σ for different materials.

Table S4: Intra-day variation for different materials.

Table S5: Inter-day variation for different materials; Table S6: Comparison Table of Different Detection Methods for *Listeria monocytogenes*

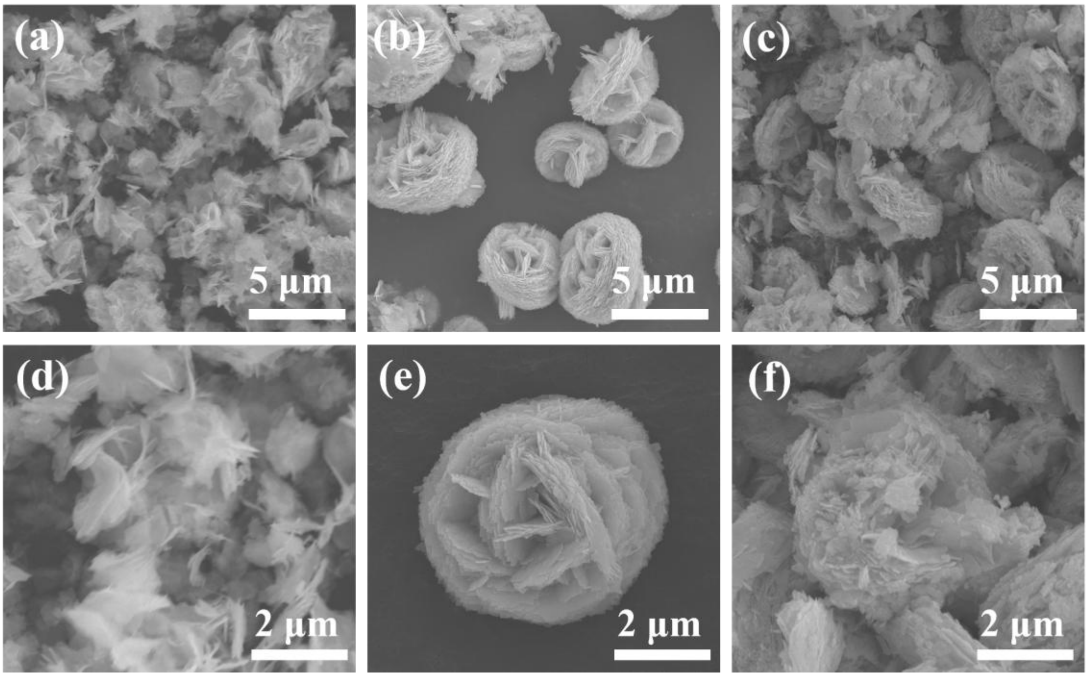

Figure S1. SEM images of Bi_2WO_6 synthesized by adding different Na_2SO_4. (a) 1g, (b) 3g, and (c) 5g.

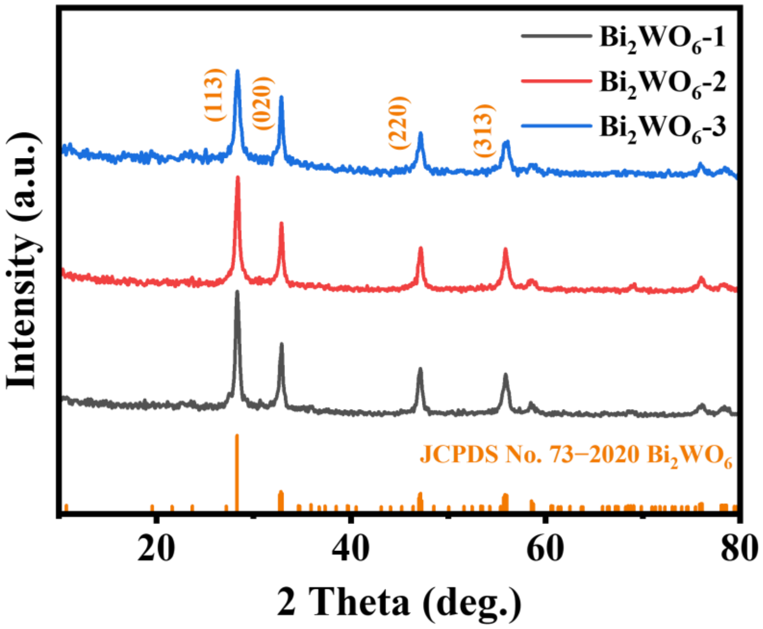

Figure S2. XRD patterns of Bi_2WO_6 with different Na_2SO_4 additions.

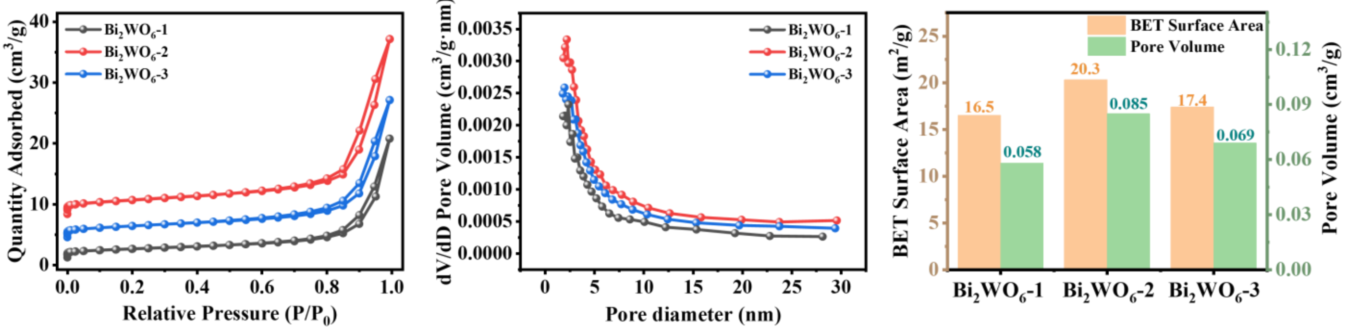

Figure S3. (a) Nitrogen adsorption-desorption isotherms, (b) pore size distributions, and (c) BET surface area and Pore volume of pristine Bi_2WO_6-1, Bi_2WO_6-2, and Bi_2WO_6-3.

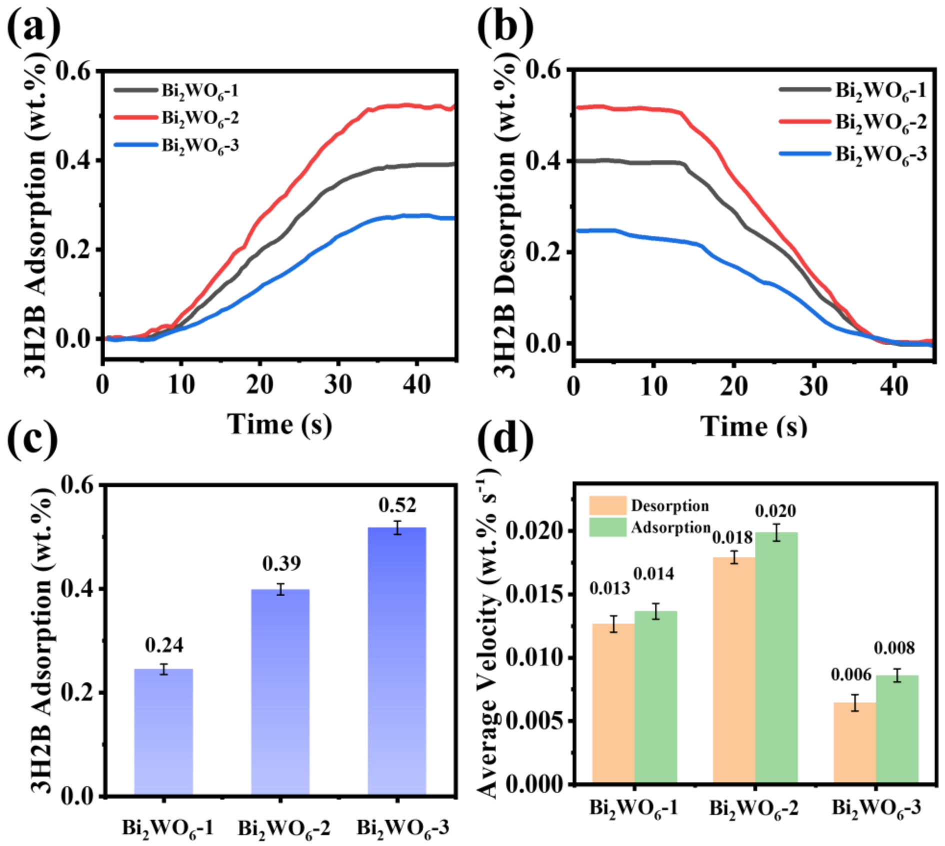

Figure S4. QCM adsorption desorption test of 3H2B on Bi_2WO_6 synthesized with different Na_2SO_4 addition amounts. (a) Adsorption curve, (b) desorption curve, (c) maximum adsorption capacity, and (d) adsorption/desorption rate.

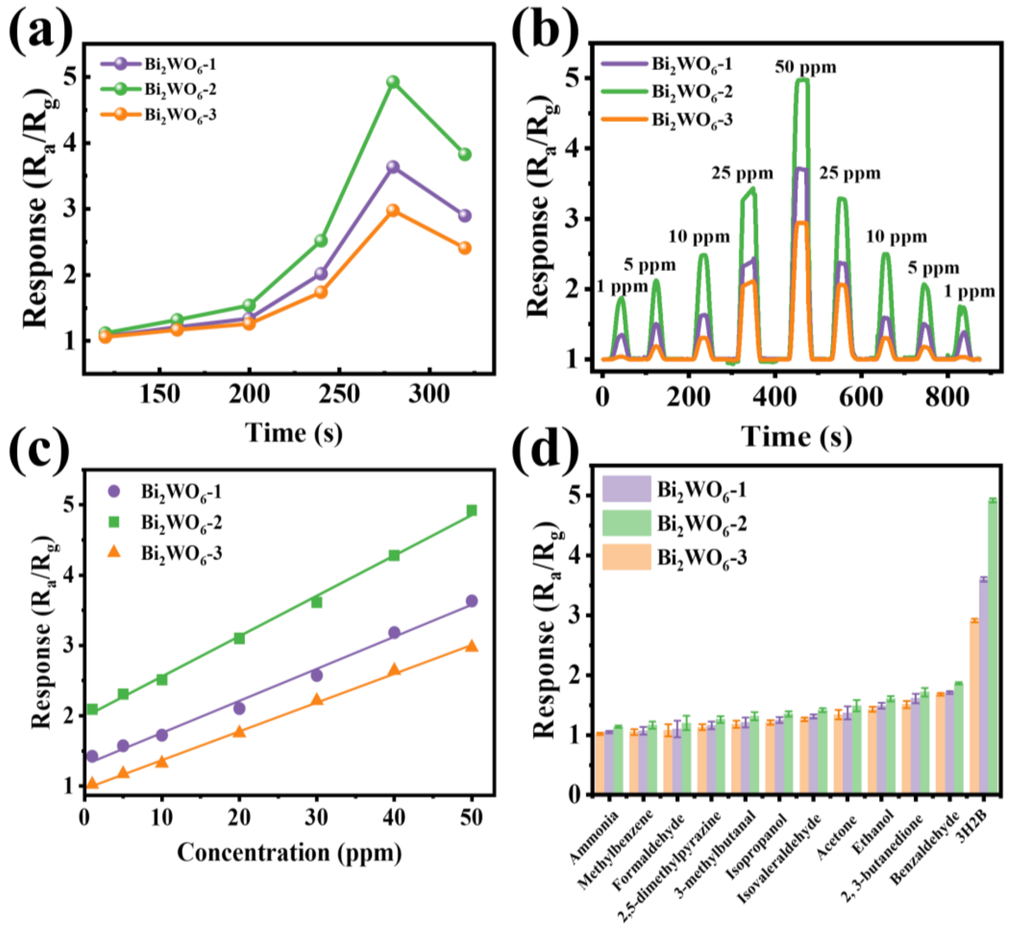

Figure S5. Gas-sensing properties of Bi_2WO_6 synthesized based on different Na_2SO_4 addition amounts for 3H2B. (a) The response of the sensor to 50 ppm 3H2B at different working temperatures (120-320 ℃). (b) Dynamic response curves of sensors to different concentrations of 3H2B (1-50 ppm) at 280 ℃. (C) Linear relationship between gas sensor response at 280 ℃ and 3H2B concentration. (d) The selectivity of the sensor towards 50 ppm 3H2B and other interfering gases at 280 ℃.

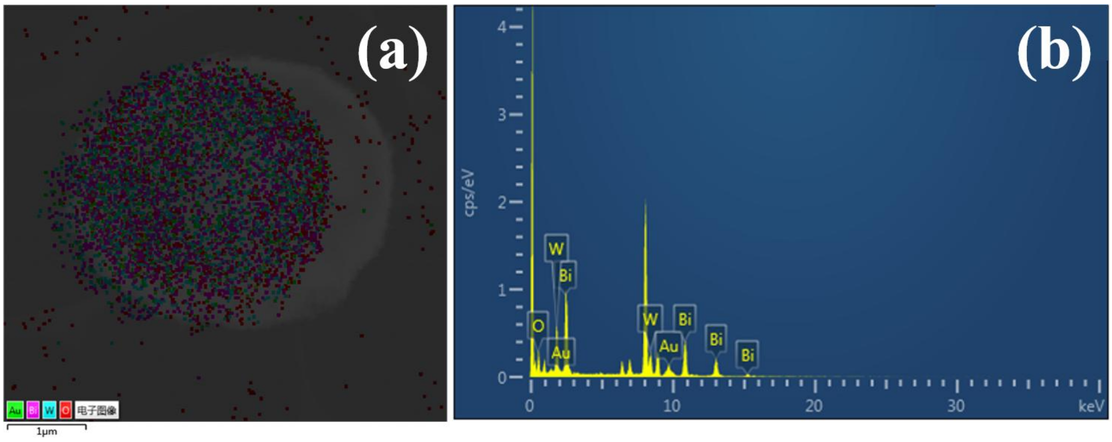

Figure S6: EDS spectra of 1.0% Au-Bi_2WO_6 material

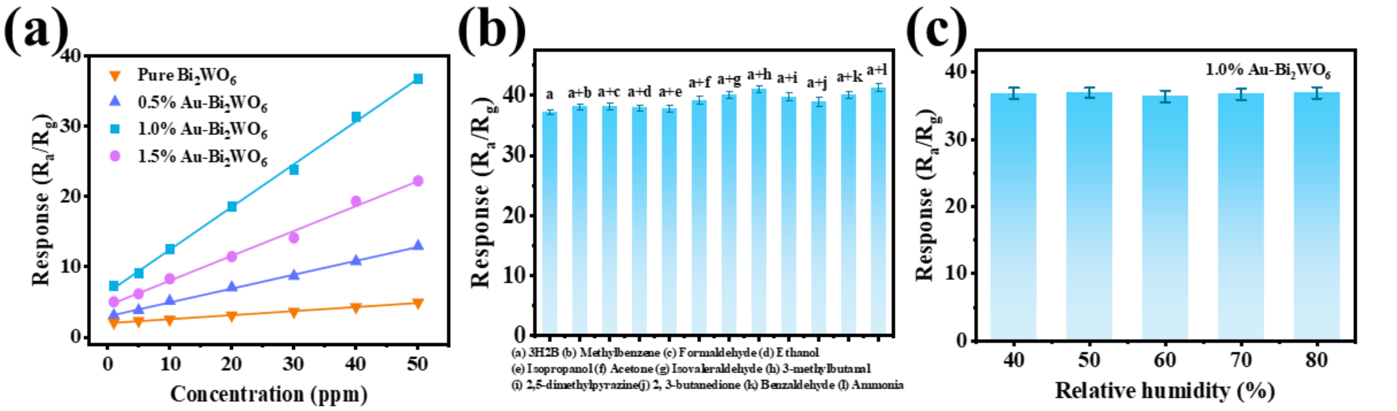

Figure S7. Gas sensing performance: (a) linear relationship between response and 3H2B concentration (1~50 ppm) of pure Bi_2WO_6, 0.5% Au-Bi_2WO_6, 1.0% Au-Bi_2WO_6, and 1.5% Au-Bi_2WO_6. (b) Response to mixed gases containing 25 ppm 3H2B and 25 ppm other interfering gases, and (c) response to 50 ppm 3H2B at different humidity values of 1.0% Au-Bi_2WO_6.

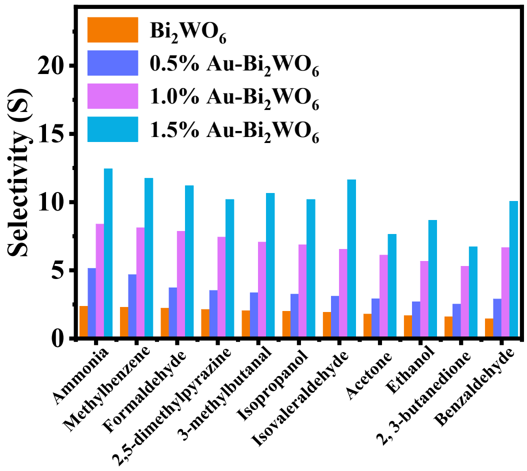

Figure S8: Selectivity coefficients of different materials for different interfering gases.

Table S1: Atomic proportion of 1.0% Au-Bi_2WO_6 material

Element	Atomic percent (%)
**O**	50.89
**W**	16.93
**Au**	1.02
**Bi**	31.16

Table S2: LOD, LOQ, RSD and Linearity range for different materials

Materials	LOD (ppb)	LOQ (ppm)	RSD (%)	Linearity range
Pure Bi_2WO_6	1173	3.91	7.7	0~50 ppm
0.5% Au-Bi_2WO_6	176	0.589	2.1	0~50 ppm
1.0% Au-Bi_2WO_6	93	0.31	0.9	0~50 ppm
1.5% Au-Bi_2WO_6	107	0.35	1.8	0~50 ppm

Table S3: Linear relationship, R^2, and σ for different materials

Materials	Linear relationship	R^2	σ
Pure Bi_2WO_6	y = 0.05724x+1.98033	0.99694	0.02239
0.5% Au-Bi_2WO_6	y = 0.19725x+2.95133	0.99824	0.01162
1.0% Au-Bi_2WO_6	y = 0.60753x+6.3444	0.99843	0.01885
1.5% Au-Bi_2WO_6	y = 0.35352x+4.48216	0.99391	0.01261

Table S4: Intra-day variation for different materials

Materials	No.1	No.2	No.3	No.4	No.5	No.6	RSD (%)
Pure Bi_2WO_6	5.04	5.09	5.12	5.01	4.93	5.08	1.35
0.5% Au-Bi_2WO_6	12.02	11.96	11.82	12.10	12.21	11.97	1.11
1.0% Au-Bi_2WO_6	36.73	36.91	37.02	37.67	36.74	36.86	0.95
1.5% Au-Bi_2WO_6	22.07	21.85	22.47	22.91	21.75	22.41	1.96

Table S5: Inter-day variation for different materials

Materials	Day1			Day2			Day3			RSD
	No.1	No.2	No.3	No.1	No.2	No.3	No.1	No.2	No.3	(%)
Pure Bi_2WO_6	5.04	5.12	5.21	5.09	5.31	4.97	5.12	5.31	5.17	2.22
0.5% Au-Bi_2WO_6	12.02	12.11	12.09	11.96	11.94	11.62	11.82	12.41	12.14	1.82
1.0% Au-Bi_2WO_6	36.73	36.42	36.12	36.91	36.45	35.98	37.02	36.95	36.74	1.01
1.5% Au-Bi_2WO_6	22.07	21.85	22.17	21.85	21.47	22.36	22.47	21.54	22.64	1.84

Table S6: Comparison Table of Different Detection Methods for Listeria monocytogenes

Technologies	Response time	LOD	Non-destructive	Ref.
CuMOF biosensor	A few minutes	6.61 CFU mL^{-1}	Destructive	[S1]
GOD@ZIF-8@Ab biosensor	A few hours	10 CFU mL^{-1}	Destructive	[S2]
Cefe-PGA-MNPs biosensor	100 min	31 CFU mL^{-1}	Destructive	[S3]
APM Fluorescence immuno-assay	1 h	3.2 CFU mL^{-1}	Destructive	[S4]
BUHNPs electrochemical sensor	7 min	1 CFU mL^{-1}	Destructive	[S5]
Amp-PEG-MBs enzyme-catalyzed sensor	66 min	10 CFU mL^{-1}	Destructive	[S6]
TFP nanozyme sensor	A few hours	10 CFU mL^{-1}	Destructive	[S7]
Fe_3O_4@MIL-100(Fe) colorimetric sensor	A few minutes	14 CFU mL^{-1}	Destructive	[S8]
CdTe/WO_3 photoelectro-chemical sensor	A few hours	45 CFU mL^{-1}	Destructive	[S9]
1.0% Au-Bi_2WO_6	13s	10 CFU mL^{-1}	Non-destructive	This work

(S1) Ye, Y.; Yan, W.; Wang, T.; et al. Dual-channel biosensor for simultaneous detection of S. typhimurium and L. monocytogenes using nanotags of gold nanoparticles loaded metal-organic frameworks. Analytica Chimica Acta 2023, 1279, 341816. DOI: 10.1016/j.aca.2023.341816

(S2) Bai, X.; Huang, J.; Li, W.; et al. Portable dual-mode biosensor based on smartphone and glucometer for on-site sensitive detection of Listeria monocytogenes. Science of the Total Environment 2023, 874, 162450. DOI: 10.1016/j.scitotenv.2023.162450

(S3) Xiao, F.; Li, W.; Wang, Z.; et al. Smartphone-assisted biosensor based on broom-like bacteria-specific magnetic enrichment platform for colorimetric detection of *Listeria monocytogenes*. Journal of Hazardous Materials 2023, 459, 132250. DOI: 10.1016/j.jhazmat.2023.132250

(S4) Servarayan, K. L.; Krishnamoorthy, G.; Sundaram, E.; et al. Optical immunosensor for the detection of *Listeria monocytogenes* in food matrixes. ACS Omega 2023, 8(18), 15979-15989. DOI: 10.1021/acsomega.3c00123

(S5) Li, Y.; Zhang, M.; Shi, X.; et al. Bacteria imprinted electrochemical sensor based on bimetallic silver-gold sea urchin-like hollow nanoparticles for ultrasensitive detection of *Listeria monocytogenes*. Microchemical Journal 2024, 205, 111206. DOI: 10.1016/j.microc.2024.111206

(S6) Bai, X.; Wang, Z.; Li, W.; et al. Portable sensor based on magnetic separation and enzyme-mediated immune nanomaterials for point-of-care testing of *Listeria monocytogenes* in food. Analytica Chimica Acta 2022, 1236, 340576. DOI: 10.1016/j.aca.2022.340576

(S7) Pan, X.; Shi, D.; Fu, Z.; et al. Rapid separation and detection of *Listeria monocytogenes* with the combination of phage tail fiber protein and vancomycin-magnetic nanozyme. Food Chemistry 2023, 428, 136774. DOI: 10.1016/j.foodchem.2023.136774

(S8) Du, J.; Li, Z.; Liu, K.; et al. Colorimetric aptasensor for Listeria monocytogenes detection using dual functional Fe_3O_4@MIL-100(Fe) with magnetic separation and oxidase-like activities in food samples. Microchimica Acta 2024, 191(8), 504. DOI: 10.1007/s00604-024-06421-9

(S9) Zhu, L.; Hao, H.; Ding, C.; et al. A novel photoelectrochemical aptamer sensor based on CdTe quantum dots enhancement and exonuclease I-assisted signal amplification for *Listeria monocytogenes* detection. Foods 2021, 10(12), 2896. DOI: 10.3390/foods10122896
